# Transcriptomic analysis of *Staphylococcus xylosus* in the presence of nitrate and nitrite in meat reveals its response to nitrosative stress

**DOI:** 10.3389/fmicb.2014.00691

**Published:** 2014-12-15

**Authors:** Aurore Vermassen, Anne de la Foye, Valentin Loux, Régine Talon, Sabine Leroy

**Affiliations:** ^1^Institut National de la Recherche Agronomique, UR454 MicrobiologieSaint-Genès-Champanelle, France; ^2^Institut National de la Recherche Agronomique, Plateforme d'Exploration du MétabolismeSaint-Genès-Champanelle, France; ^3^Institut National de la Recherche Agronomique, UR1077 Mathématique, Informatique et GénomeJouy-en-Josas, France

**Keywords:** *Staphylococcus xylosus*, starter, nitrate, nitrite, transcriptome, nitrosative stress, meat

## Abstract

*Staphylococcus xylosus* is one of the major starter cultures used for meat fermentation because of its crucial role in the reduction of nitrate to nitrite which contributes to color and flavor development. Despite longstanding use of these additives, their impact on the physiology of *S. xylosus* has not yet been explored. We present the first *in situ* global gene expression profile of *S. xylosus* in meat supplemented with nitrate and nitrite at the levels used in the meat industry. More than 600 genes of *S. xylosus* were differentially expressed at 24 or 72 h of incubation. They represent more than 20% of the total genes and let us to suppose that addition of nitrate and nitrite to meat leads to a global change in gene expression. This profile revealed that *S. xylosus* is subject to nitrosative stress caused by reactive nitrogen species (RNS) generated from nitrate and nitrite. To overcome this stress, *S. xylosus* has developed several oxidative stress resistance mechanisms, such as modulation of the expression of several genes involved in iron homeostasis and in antioxidant defense. Most of which belong to the Fur and PerR regulons, respectively. *S. xylosus* has also counteracted this stress by developing DNA and protein repair. Furthermore, it has adapted its metabolic response—carbon and nitrogen metabolism, energy production and cell wall biogenesis—to the alterations produced by nitrosative stress.

## Introduction

Staphylococci are commensals of the skin and mucous membranes of animals and are found in various niches (Kloos et al., 1976; Nagase et al., 2002). Products of animal origin are naturally contaminated by staphylococci. *Staphylococcus xylosus* is one of the three species frequently isolated from cheeses and dry fermented sausages (Coton et al., 2010). Furthermore, *S. xylosus* is commonly used as starter culture in meat fermentation (Talon and Leroy, 2011). This bacterium contributes to the development of flavor through its antioxidant properties and degradation of amino acids (Barrière et al., 2001a,b, 2002; Talon et al., 2002). But its main function in cured products is to reduce nitrate to nitrite which is necessary for the development of color and flavor. In fermented meat products, nitrite is then reduced by chemical reactions to nitric oxide (NO), which interacts with myoglobin to form nitrosomyoglobin, which gives the meat a stable red color (Gøtterup et al., 2007). Nitrite is responsible for the production of the characteristic flavor of cured meat through complex mechanisms not entirely understood. Furthermore, nitrite and derived compounds act in meat as an antioxidant preventing lipid oxidation and therefore the development of rancid off-flavors (Cammack et al., 1999). Besides its role in color and flavor formation, nitrite preserves meat products against growth of undesirable anaerobic bacteria and some pathogenic bacteria (Tompkin, 1995).

The reduction of nitrate and nitrite is well described in laboratory media for *Staphylococcus carnosus*, another meat starter culture (Neubauer and Götz, 1996). Compared with *S. carnosus*, the nitrate reductase activity of *S. xylosus* is poorly characterized and the nitrite reductase has not been described. Most strains of *S. xylosus*, like other meat-associated staphylococci, have a nitrate reductase activity (García-Varona et al., 2000; Mauriello et al., 2004).

Cured meat products have been manufactured using nitrate and nitrite since ancient times. In most countries, the use of nitrate and nitrite, usually added as potassium or sodium salts, is controlled in cured meat. Added or residual amounts are regulated by laws. A European parliament and council directive (directive 2006/52/EC) established limits of 150 mg nitrite/kg and 300 mg nitrate/kg for non-heated meat products. The limits on residual amounts are 50 mg nitrite/kg and 250 mg nitrate/kg in dried meat products (Honikel, 2008). Nitrate (after its conversion to nitrite) and nitrite have various valuable properties, but their safety is still questioned because of their contribution to the formation of carcinogenic nitrosamines. The current aim is to further restrict the use of nitrate and nitrite in meat products. Functional starter cultures may be useful to reduce the levels of nitrate and nitrite as suggested by Leroy et al. (2006). However, the impact of these curing agents on the physiology of the starter cultures in meat is still unknown.

In this study, we characterized the transcriptomic effects of nitrate and nitrite at the levels used in the meat industry on the physiology of *S. xylosus* directly in a meat model, thereby establishing for the first time the *in situ* response to these additives. The transcriptome was analyzed in meat incubated in conditions that mimic the fermentation step in sausage manufacturing. The nitrosative stress generated by addition of nitrate and nitrite may trigger defense mechanisms developed by *S. xylosus*. One concerns modulation of the expression of several genes involved in iron homeostasis and the other concerns genes involved in antioxidant defense. Furthermore, this stress modifies the expression of several genes involved in carbon and nitrogen metabolism, energy production, cell wall biogenesis, and DNA and protein repair.

## Materials and methods

### Bacterial strain and culture conditions

The *S. xylosus* C2a strain is derived from the type strain DSM20267 cured of its endogenous plasmid pSX267. We used this laboratory strain as it is the only one with some genetic background. Moreover, its complete genome has been sequenced (LN554884). The strain was cultured overnight at 30°C in a minimal medium (Fiegler and Brückner, 1997). The culture was centrifuged and the cell pellet was resuspended in physiological serum and used to inoculate the meat model with 10^7^ CFU/g of meat. To evaluate the inoculum and growth of *S. xylosus* in meat, bacteria were enumerated after serial dilutions on plates of brain-heart infusion agar (Difco Laboratories, Detroit, MI), which were incubated at 30°C for 24 h.

### Meat model

Frozen vacuum-packed ground pork meat sterilized by irradiation (15 kGy) was used. After thawing the meat at 4°C, two batches were prepared. One batch with nitrate and nitrite was prepared as follows per kg of meat: 5 g of glucose, 27.7 g of NaCl, 0.18 g of KNO_3_, 0.03 g of NaNO_2_. The second batch was prepared as before but without nitrate and nitrite. After inoculation with *S. xylosus*, each batch was distributed in glass Petri dishes and incubated at 22°C in a wet atmosphere for up to 72 h. Three independent experiments were done.

### Analytical methods

The samples were analyzed at 6 times of incubation (0, 2, 8, 24, 48, and 72 h): measurement of meat pH (pH meter MP230, Mettler Toledo/ sonde Inlab 413,Viroflay, France), evaluation of nitrate and nitrite concentrations in meat by reducing nitrate to nitrite with metallic cadmium and by addition of sulphanilamide chloride and N-(1-naphthyl)-ethylene-diamine dihydrochloride plus measurement of absorbance at 538 nm (NF V04-410), and visual evaluation of meat surface color.

### Total RNA extraction

At 24 and 72 h of incubation, several aliquots of 200 mg meat samples were taken from each batch of the three independent experiments and immediately frozen in liquid nitrogen to stabilize the bacterial RNA. For RNA extraction, 1.25 mL of Trizol reagent (Invitrogen, Cergy Pontoise, France) was added to the meat sample. The samples were transferred to tubes containing 800 mg of zirconia-silica beads (0.1 mm diameter) used to break the bacterial cells and were vigourously shaken in a bead beater (FastPrep, MP Biomedicals, Illkirch-Graffenstaden, France), after which 280 μL of chloroform was added. After centrifugation, the upper phase containing RNA was collected and a mixture of acid phenol/chloroform/isoamyl alcohol (25/24/1) was added. After centrifugation, the upper phase was purified with the Nucleospin RNA II kit (Macherey Nagel, Hoerdt, France) according to the manufacturer's instructions. A supplementary treatment was performed with Turbo DNAse (Ambion, Austin, TX) to remove all traces of contaminating DNA. The absence of *S. xylosus* genomic DNA contamination was verified by PCR targeting the *rpoB* gene. Total RNA isolated was quantified using a Nanodrop 1000 (Thermo Fisher Scientific, Wilmington, DE) and RNA quality was analyzed using an Agilent 2100 Bioanalyzer (Agilent Technologies, Santa Clara, CA) according to the manufacturer's instructions. The RNA was stored at −80°C.

### RNA labeling and microarray

We have developed a DNA microarray specific to *S. xylosus* C2a based on an Agilent technology microarray. This array has 19,805 probes targeting the whole *S. xylosus* C2a genome. On average there are 6–10 probes per gene. A complete description of the array is available at the NCBI Gene Expression Omnibus (GEO) database under platform accession number GPL19201.

RNA from each sample was reverse transcribed to cDNA with SuperScript Reverse Transcriptase according to the manufacturer's instructions (Invitrogen). The RNA-cDNA hybrids were then digested using RNAse H (Invitrogen). The cDNA was labeled with Cy3 dCTP or Cy5 dCTP using the Bioprime DNA kit according to the manufacturer's instructions (Invitrogen). Unincorporated Cy-dye was removed using a CentriSep column (Applied Biosystems, Warrington, United Kingdom) and dye swap experiments were performed. Cy3/cDNA and Cy5/cDNA were mixed with Agilent hybridization buffer and hybridized at a temperature of 60°C for 17 h in a dedicated hybridization oven. After washing and drying, microarrays were scanned in a SYS-SN-ARRAY Agilent Microarray Scanner (5-μm resolution) with a coefficient of photomultiplication of 30%.

### Microarray data analysis and statistical treatment

Microarrays were analyzed by the Feature extraction software (Agilent, version 9.1.3.1) in 3 steps: localization of the spots, segmentation of the pixels and extraction of qualitative and quantitative data. These data were filtered to remove spots that were saturated or of poor quality. Within each array, Cy3/Cy5 log-ratios were normalized to correct for the mean spot intensity-dependent dye effect. Significant differences in the probe set intensities between the two conditions were identified using a linear model with an empirical Bayes method using all information probes to moderate the standard errors of the estimated log-fold changes (Smith, 2004). The probabilities were corrected by the Benjamini-Hochberg procedure in order to control the false-discovery rate (FDR) with a *p*-value cut-off of 0.05. All the probes with an FDR ≤ 0.05 are considered to be differentially expressed. Finally, one gene was considered to be differentially expressed if at least 66% of the corresponding probes were differentially expressed.

### Quantitative PCR (qPCR)

qPCR was used to validate the microarray analysis and was performed using Real Plex Master Cycler (Eppendorf, Hamburg, Germany) with IQ™ SYBR®Green Supermix (Bio-Rad, Hercules, CA). Thermal cycling consisted of 30 s at 95°C, followed by 40 cycles of 15 s at 95°C and 60 s at 60°C. The targeted genes and primer sequences are listed in Supplementary Table 1. All genes were quantified in duplicate for the three independent experiments. The analyses were performed on the same batches of RNA as those used for the microarray experiments. The housekeeping gene *rpoB* was used as reference gene for normalization. Results were calculated using the comparative cycle threshold method (Pfaffl, 2001).

### Microarray data accession number

The microarray samples and data have been deposited in the GEO database under accession number GSE61514.

## Results and discussion

### Behavior of *S. xylosus* in the meat model

Bacterial growth was similar in the two batches of meat with or without nitrate and nitrite in the three independent replicates. Inoculated at 5 × 10^7^ CFU/g, the growth of *S. xylosus* was exponential until t_24h_ when the population reached 9 × 10^8^ CFU/g. It remained in the stationary phase until the end of the experiment (Supplementary Figure 1). These results differ from those of Neubauer and Götz (1996) showing that nitrate and nitrite promote the growth of *S. carnosus*. However, Neubauer and Götz's experimental conditions differed greatly from ours: *in vitro* in laboratory media vs. *in situ* in our meat model, and nitrate (25 mM) and nitrite (2 mM) concentrations that were very high compared with the 0.18 mM and 0.09 mM, respectively, we used.

The pH was measured at each point between t_0h_ and t_72h_. As expected, only a weak acidification was observed in the meat model. The pH values were near 5.9 at t_0h_ and near 5.7 at t_72h_. Addition of nitrate and nitrite had no effect on acidification in the meat. In our experiments, pH was less acid than in fermentation carried out in the presence of lactic acid bacteria, in which the pH can range from 4.5 to 5.3 (Demeyer et al., 2000).

Nitrate and nitrite were not detected in the batch prepared without nitrate and nitrite, whereas the initial concentrations of nitrate (180 mg/kg of meat) and nitrite (30 mg/kg of meat) added to the meat model were found at t_0h_. Nitrate concentration decreased to 31 mg/kg of meat until t_24h_ and remained at 25 mg/kg until t_72h_. Concomitantly, the nitrite concentration rose to 133 mg/kg of meat at t_24h_ and then decreased slowly to 92 mg/kg of meat at t_72h_.

In parallel, a sharp color difference was observed between the two batches. In the presence of nitrate and nitrite at t_72h_, the meat was red because of the formation of nitrosylmyoglobin (MbFeIINO), while the meat in the other batch was brown due to the production of metmyoglobin (data not shown). In meat, the nitrite undergoes chemical reactions that lead to reactive nitrogen species (RNS), including NO (Honikel, 2008; Hammes, 2012). These RNS as well as reactive oxygen species (ROS) can interact with and damage numerous targets, including thiols, metal centers, tyrosine residues, nucleotide bases and lipids (Gaupp et al., 2012).

### Transcriptomic analysis and validation

A total of 627 genes were differentially expressed at t_24h_ and t_72h_ in the presence of nitrate and nitrite *in situ* in meat. This represented close to 24% of the 2634 total predicted genes in strain C2a. There were 466 genes at t_24h_ with 222 up- and 244 down-regulated and 241 genes at t_72h_ with 112 up- and 129 down-regulated. Only 80 common genes were differentially expressed at the two times of incubation. All the genes that passed our selection criteria are listed in Supplementary Table 2.

**Figure 1:**
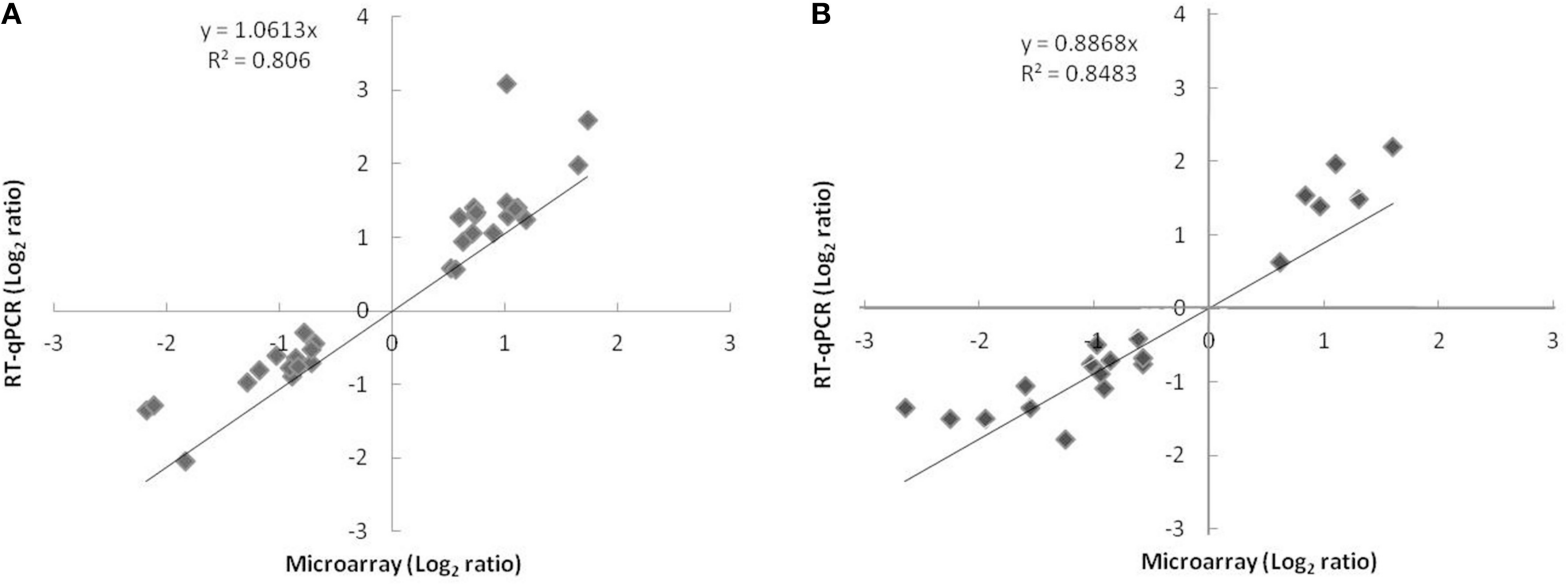
**Comparison of log2 expression ratios of 38 differentially regulated genes measured by using microarray and qPCR at t_24h_ (A) or t_72h_ (B)**. Positive and negative log2 expression ratios represent up- and down-regulation in the presence of nitrate and nitrite. Each data point is calculated from averages of biological triplicates.

To validate the microarray analysis independently, the relative expression of 38 differentially expressed genes representing 6% of genes with significantly modified expression was measured by qPCR. The microarray and qPCR results for the tested genes were strongly correlated at 24 h (*r*^2^ = 0.806; slope = 1.061) and 72 h (*r*^2^ = 0.848; slope = 0.886) and the expected trend in the expression pattern was confirmed (Figure 1).

### COG distribution in the transcriptome

**Figure 2:**
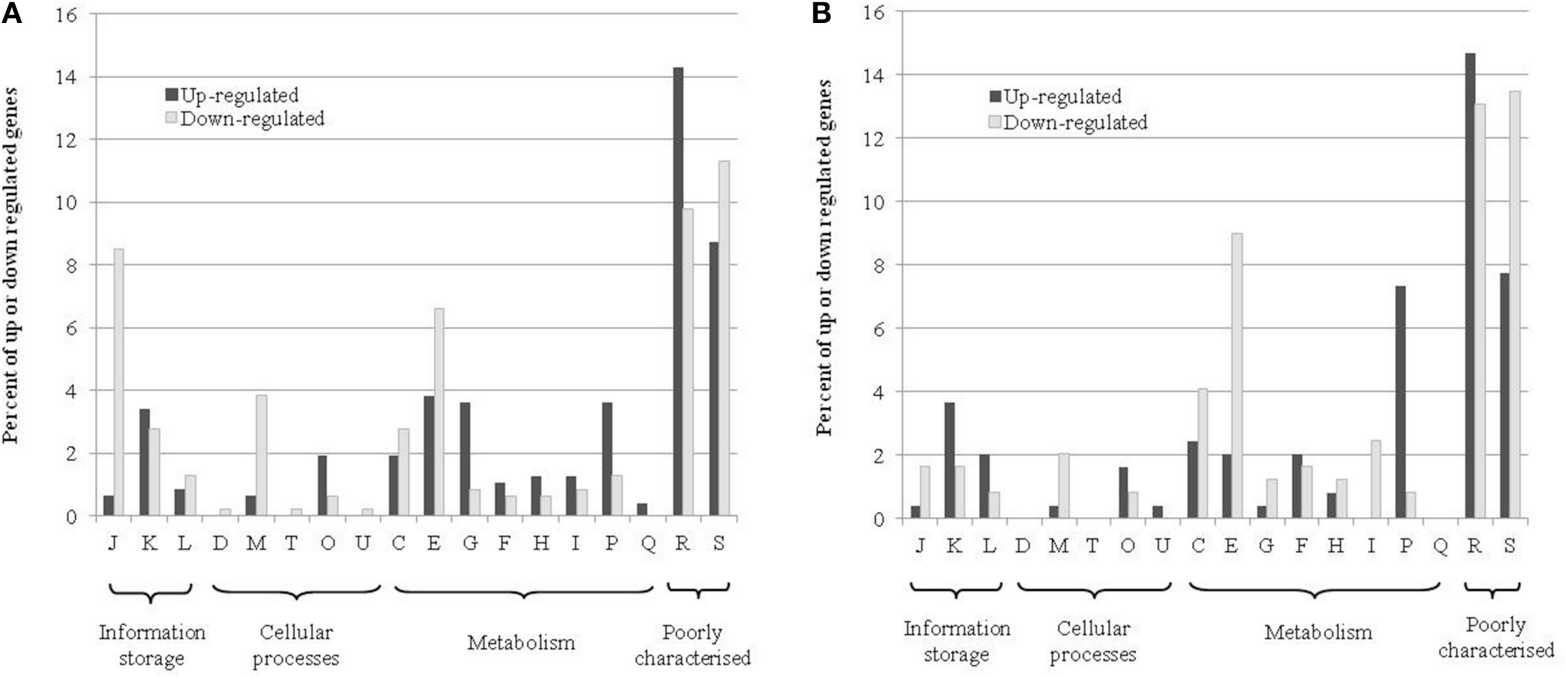
**COG distribution of differentially up-and down-regulated genes in the transcriptome at t_24h_ (A) or t_72h_ (B)**. The percentages represent the number of genes that are in a category relative to the respective total number of genes that were up- or down-regulated. The COG abbreviations are as follows: [J] translation, ribosomal structure, and biogenesis; [K] transcription; [L] replication, recombination, and repair; [D] cell cycle control, cell division, and chromosome partitioning; [M] cell wall/membrane/envelope biogenesis; [T] signal transduction mechanisms; [O] post-translational modification, protein turnover, and chaperones; [U] intracellular trafficking, secretion, and vesicular transport; [C] energy production and conversion; [E] amino acid transport and metabolism; [G] carbohydrate transport and metabolism; [F] nucleotide transport and metabolism; [H] coenzyme transport and metabolism; [I] lipid transport and metabolism; [P] inorganic ion transport and metabolism; [Q] secondary metabolite biosynthesis, transport, and catabolism; [R] general function prediction only; [S] function unknown.

The COG distribution of genes up- or down-regulated in response to RNS generated from nitrate and nitrite is shown in Figure 2. The most represented groups are genes involved in information storage and processing, cellular processes and metabolism. Many of the differentially regulated genes are unclassified or belong to the category “function unknown” [S] or “general function prediction only” [R]. At t_24h_, for the up-regulated genes, the most-well represented categories were transcription [K], post-translational modification, protein turnover, and chaperones [O], amino acid transport and metabolism [E], carbohydrate transport and metabolism [G], inorganic ion transport and metabolism [P]. For the down-regulated genes, the 4 dominant categories were cell wall/membrane/envelope biogenesis [M], energy production and conversion [C], amino acid transport and metabolism [E] and translation, ribosomal structure, and biogenesis [J]. We observed a similar distribution for up- and down-regulated genes at t_72h_.

### Expression of *nar* and *nir* genes

As in fermented meat products, in our experiment nitrate was reduced to nitrite by *S. xylosus*. The organization of *nar* and *nir* loci in the *S. xylosus* C2a genome is the same as in *S. carnosus*. No differential expression of *nar* and *nir* genes was noted between the two batches, i.e., with or without nitrate and nitrite at t_24h_ and t_72h_. This is not surprising, at least for *nar* expression, as the reduction of nitrate to nitrite was mostly achieved at t_24h_, as revealed by the assays of nitrate and nitrite during the incubation. Furthermore, the nitrate reductase activity of *S. xylosus* peaked during exponential growth in laboratory media supplemented with nitrate (Talon et al., 1999). The nitrite can modulate the transcription of the *nir* operon during anaerobic and aerobic growth of *S. carnosus* (Neubauer et al., 1999). But in this *in vitro* study, 2 mM nitrite was used to enhance the transcriptional level. In our conditions, the maximum concentration of nitrite measured was 0.4 mM. In *S. carnosus*, the transcription of the *nar* and *nir* operons is under the control of NreBC, which regulates anaerobic respiration (Fedtke et al., 2002). In our meat model, with or without nitrate and nitrite, *S. xylosus* is subject to the same oxygen conditions, which could explain the non-differential expression of *nar* and *nir* genes.

### Iron homeostasis and the fur regulon

In meat, *S. xylosus* responded to the presence of RNS by the up-regulation of 17 genes involved in iron acquisition (Table 1). Most of these genes were related to the production or harnessing of siderophores (*sfa, hts, fhu, sst*). Two siderophores may be produced by staphylococci: staphyloferrin A (SA) and staphyloferrin B (SB) (Beasley and Heinrichs, 2010; Sheldon and Heinrichs, 2012; Saha et al., 2013). In our study, only the genes *sfaABCD* with genetic organization similar to that of *Staphylococcus aureus* encoding enzymes for the synthesis of SA were identified (Hammer and Skaar, 2011). Adjacent to this locus, also as described in *S. aureus*, a cluster of 3 genes *htsABC* encodes the ABC transporter for uptake of Fe-SA. The *hts-sfa* locus is found in all staphylococcal genomes, while the locus encoding SB is only found in *S. aureus* and *Staphylococcus pseudointermedius* (Sheldon and Heinrichs, 2012). In parallel, we confirmed by a chrome azurol S assay that the *S. xylosus* strain C2a incubated in Staphylococcal Siderophore Detection medium (Schwyn and Neilands, 1987) produced siderophores (data not shown). Staphylococci have not been shown to synthesize hydroxamate and catechol siderophores, but they are known to be able to utilize them as iron sources (Sheldon and Heinrichs, 2012). *S. xylosus* possesses both the Fhu system involved in the uptake of hydroxamate- and the Sst system for the catechol-type siderophores. As for *S. aureus*, a cluster of three genes *fhuCBG* encodes a dimeric permease (FhuBG) and an ATPase (FhuC) and two separate genes (*fhuD1, fhuD2*) encode substrate-binding lipoproteins. FhuC serves also as an ATPase for the import of Fe-SA (Sheldon and Heinrichs, 2012). Finally, a cluster of four genes *sstABCD* encodes an ABC transporter and SstD, which binds ferrated catechol siderophores and catecholamines, but also a broader range of potential substrates. This locus is highly conserved in *S. aureus* strains and is present in most coagulase-negative staphylococci (Sheldon and Heinrichs, 2012). The single gene *isdG* encoding a cytoplasmic heme-degrading monooxygenase was up-regulated in *S. xylosus* in the presence of RNS. In *S. aureus*, this gene belongs to the operon *isdCDEFsrtBisdG* which, together with four other operons, encodes the Isd system involved in heme-mediated iron acquisition (Hammer and Skaar, 2011). Unlike *S. aureus, S. xylosus* does not possess the other genes of the Isd system. Heme is used as a cofactor in many biological systems due to the redox potential of the iron atom, but at high levels this molecule can be toxic. IsdG could be used to regulate endogenous heme synthesis and metabolism (Haley and Skaar, 2012). Degradation of heme by IsdG releases iron, carbon monoxide and oxo-bilirubin, which has potent antioxidant properties (Haley and Skaar, 2012). IsdG has been found in many classes of bacteria, which underlines the importance of heme degradation. Despite the role of heme as cofactor and as iron and bilirubin sources, heme homeostasis is not yet understood (Haley and Skaar, 2012).

**Table 1:** **Genes of *Staphylococcus xylosus* discussed in this study differentially expressed over time in meat in the presence of nitrate and nitrite**.

**Functional category and family**	**Gene ID**	**Gene name**	**Protein**	**Mean ratio of expression**	
				**24 h**	**72 h**
**P: INORGANIC ION TRANSPORT AND METABOLISM**					
Iron	SXYL_02116	*sstA*	Iron compound ABC transporter, permease protein SstA	2.8	3.3
	SXYL_02115	*sstB*	Iron compound ABC transporter, permease protein SstB	3.1	5.3
	SXYL_02114	*sstC*	Iron compound ABC transporter, ATP-binding protein SstC	2.5	3.9
	SXYL_02113	*sstD*	Lipoprotein SstD	2.5	3.8
	SXYL_02202	*fhuB*	ABC-type cobalamin Fe^3+^-siderophores transport system permease component	2.6	1.8
	SXYL_02203	*fhuC*	ABC-type cobalamin Fe^3+^-siderophores transport system ATPase component	2.7	
	SXYL_02201	*fhuG*	ABC-type cobalamin/Fe^3+^-siderophores transport systems, permease components	2.2	1.8
	SXYL_00667	*fhuD1*	Iron^(3+)^-hydroxamate-binding protein	2.3	1.8
	SXYL_02681	*fhuD2*	Iron^(3+)^-hydroxamate-binding protein	2.0	1.8
	SXYL_00748	*sfaA*	Transporter SfaA	1.9	1.6
	SXYL_00749	*sfaB*	Siderophore biosynthesis protein, IucA/IucC family	1.7	1.4
	SXYL_00750	*sfaC*	amino acid racemase	1.5	1.3
	SXYL_00747	*sfaD*	Siderophore biosynthesis protein, IucA/IucC family	2.4	2.0
	SXYL_00751	*htsA*	Iron compound ABC transporter, iron compound-binding protein	3.1	2.5
	SXYL_00752	*htsB*	Iron compound ABC transporter, permease protein	1.7	2.0
	SXYL_00753	*htsC*	Iron compound ABC transporter, permease protein	1.7	1.9
	SXYL_00755	*isdG*	Heme-degrading monooxygenase	2.0	
	SXYL_00944	*ftnA*	Ferritin	1.6	
Manganese	SXYL_01831	*mntH*	Divalent metal cation transporter MntH	1.5	2.3
Magnesium	SXYL_01923	*mgtE*	Divalent cation transporter MgtE	0.7	
**O: POST-TRANSLATIONAL MODIFICATION, PROTEIN TURNOVER, CHAPERONES**					
Antioxidant defenses	SXYL_02505	*katA*	Catalase A	0.3	0.6
	SXYL_01551	*katB*	Catalase B	2.3	
	SXYL_02533	*katC*	Catalase C	2.3	
	SXYL_02534	*ahpC*	Alkyl hydroperoxide reductase subunit C		2.0
	SXYL_02083	*trxB*	Thioredoxin reductase	1.6	
	SXYL_00973	*bcp*	Bacterioferritin comigratory protein	2.0	
	SXYL_01572	*bsaA*	Glutathione peroxidase	2.2	
	SXYL_00570		Ferredoxin–NADP reductase	2.4	1.9
	SXYL_00895		Nitroreductase family protein		1.8
	SXYL_02021		Nitroreductase family protein	2.1	
	SXYL_00410		Putative NAD(P)H nitroreductase	1.9	
	SXYL_01517	*msrA1*	Peptide methionine sulfoxide reductase MsrA 1	1.5	
Chaperones	SXYL_00898	*groS*	10 kDa chaperonin		2.0
	SXYL_02418	*hslO*	33 kDa chaperonin	1.9	
	SXYL_02396	*clpC*	ATP-dependent Clp protease ATP-binding subunit ClpC		2.1
**G: CARBOHYDRATE TRANSPORT AND METABOLISM**					
	SXYL_02255		Phosphotransferase system (PTS) maltose-specific enzyme IICB component	2.3	
	SXYL_00253		PTS system, glucose-specific IIBC component	0.5	
	SXYL_00278		PTS system, fructose-specific IIABC components	3.4	
	SXYL_00626		PTS system arbutin-like IIBC component	1.5	
	SXYL_01351	*malA*	Alpha-D-1,4-glucosidase	1.9	
	SXYL_01960	*pgi*	Glucose-6-phosphate isomerase	2.2	
	SXYL_00426	*fbp*	Fructose-1,6-bisphosphatase	1.8	
	SXYL_00039	*iolJ*	Fructose-bisphosphate aldolase, class II		1.9
	SXYL_00221	*fda*	Fructose-bisphosphate aldolase class 1	2.5	
	SXYL_01518	*rbsB*	Ribose ABC transporter substrate-binding protein	2.2	
	SXYL_01519	*rbsC*	Ribose ABC transporter permease	2.3	
	SXYL_01521	*rbsD*	D-ribose pyranase	2.1	
	SXYL_00125	*araA*	L-arabinose isomerase		0.4
	SXYL_00124	*araD*	L-ribulose-5-phosphate 4-epimerase		0.4
	SXYL_00126	*araT*	Arabinose-proton symporter		0.6
	SXYL_00607	*araB1*	Ribulokinase	2.0	
	SXYL_02331	*araB2*	Ribulokinase		0.5
**E: AMINO ACID TRANSPORT AND METABOLISM**					
Valine, leucine, isoleucine	SXYL_02474	*brnQ1*	Branched-chain amino acid transport system II carrier protein	0.3	0.5
	SXYL_00871	*leuA*	2-isopropylmalate synthase	0.2	0.2
	SXYL_00870	*leuB*	3-isopropylmalate dehydrogenase	0.2	0.2
	SXYL_00869	*leuC*	3-isopropylmalate dehydratase large subunit	0.2	0.2
	SXYL_00868	*leuD*	3-isopropylmalate dehydratase small subunit	0.2	0.3
	SXYL_00867	*ilvA*	L-threonine dehydratase biosynthetic IlvA	0.3	0.3
	SXYL_00874	*ilvB*	Acetolactate synthase	0.3	0.2
	SXYL_00872	*ilvC*	Ketol-acid reductoisomerase	0.2	0.2
	SXYL_00875	*ilvD1*	Dihydroxy-acid dehydratase	0.3	0.2
Glycine, serine, threonine	SXYL_01317	*gcvT*	Aminomethyltransferase	3.5	
	SXYL_01318	*gcvPA*	Probable glycine dehydrogenase [decarboxylating] subunit 1	3.6	
	SXYL_01319	*gcvPB*	Probable glycine dehydrogenase [decarboxylating] subunit 2	3.0	
	SXYL_02528	*sdaAA1*	L-serine dehydratase, iron-sulfur-dependent, alpha subunit	0.6	
	SXYL_02529	*sdaAB1*	L-serine dehydratase subunit beta	0.5	
Methionine	SXYL_00012		Homoserine O-acetyltransferase		0.5
Tryptophan, phenylalanine, tyrosine	SXYL_01497	*trpA*	Tryptophan synthase alpha chain	0.6	0.5
	SXYL_01498	*trpB*	Tryptophan synthase beta chain		0.4
	SXYL_01500	*trpC*	Indole-3-glycerol phosphate synthase	0.6	0.4
	SXYL_01501	*trpD*	Anthranilate phosphoribosyltransferase	0.5	0.4
	SXYL_01502	*trpG*	Anthranilate synthase component II	0.5	0.4
	SXYL_01503	*trpE*	Anthranilate synthase component I	0.5	0.4
	SXYL_01128		DAHP synthetase-chorismate mutase		0.5
	SXYL_01513	*tyrA*	Prephenate dehydrogenase		3.1
	SXYL_00922	*pheA*	Prephenate dehydratase		0.6
	SXYL_02022	*aroD*	3-dehydroquinate dehydratase	1.7	
	SXYL_01261	*aroE*	Shikimate dehydrogenase	0.6	
	SXYL_01316	*aroK*	Shikimate kinase		0.5
Urea, arginine, aspartate	SXYL_00296	*ureA*	Urease gamma subunit	3.1	
	SXYL_00294	*ureC*	Urease subunit alpha	3.3	
	SXYL_00290	*ureD*	Urease accessory protein UreD	3.0	
	SXYL_00291	*ureG*	Urease accessory protein UreG	3.0	
	SXYL_00293	*ureE*	Urease accessory protein ureE	2.6	
	SXYL_00292	*ureF*	Urease accessory protein UreF	2.7	
	SXYL_00238	*rocD1*	Ornithine aminotransferase 1	0.6	
	SXYL_00241	*argB*	Acetylglutamate kinase	0.6	
	SXYL_00239	*argC*	N-acetyl-gamma-glutamyl-phosphate reductase	0.6	
	SXYL_02450		Arginine/lysine/ornithine decarboxylase	0.7	
	SXYL_01961	*argG*	Argininosuccinate synthase		1.6
	SXYL_01373	*asnA*	L-asparaginase	1.9	
Peptidase	SXYL_01980	*ampA*	Cytosol aminopeptidase	1.9	
	SXYL_01931		Oligoendopeptidase F	1.7	
	SXYL_01348		Peptidase T-like protein	1.6	
	SXYL_00377	*dapE*	Probable succinyl-diaminopimelate desuccinylase	2.3	
Glycine, betaine, carnitine, choline	SXYL_00488	*opuCA*	Glycine betaine/carnitine/choline ABC transporter	0.3	
	SXYL_00489	*opuCB*	ABC-type proline glycine betaine transport system permease component	0.3	
	SXYL_00490	*opuCC*	Glycine betaine/carnitine/choline ABC transporter opuCC	0.3	1.8
	SXYL_00491	*opuCD*	ABC-type proline glycine betaine transport system permease component	0.3	2.0
	SXYL_01535	*opuD1*	Glycine betaine transporter	0.5	0.5
**C: ENERGY PRODUCTION AND CONVERSION**					
	SXYL_00830	*atpD*	ATP synthase subunit beta	0.7	
NA^(+)^/H^(+)^ antiporter	SXYL_01976	*mnhG1*	Na^(+)^/H^(+)^ antiporter subunit G1		1.4
	SXYL_02226	*mnhA2*	Putative antiporter subunit A2	0.6	1.3
	SXYL_02223	*mnhD2*	Putative antiporter subunit D2	0.5	
	SXYL_02222	*mnhE2*	Putative antiporter subunit E2	0.5	
	SXYL_02221	*mnhF2*	Putative antiporter subunit F2	0.5	
	SXYL_02220	*mnhG2*	Putative antiporter subunit G2	0.6	
	SXYL_00623		Putative Na^(+)^/H^(+)^ antiporter	0.5	0.3
	SXYL_00425		Na^(+)^/H^(+)^ exchanger	0.6	
	SXYL_00363	*nhaC*	Na^(+)^/H^(+)^ antiporter NhaC	2.0	2.2
**M: CELL WALL/MEMBRANE BIOGENESIS**					
	SXYL_01990	*dltA*	D-alanine—poly(phosphoribitol) ligase subunit 1	0.3	0.7
	SXYL_01989	*dltB*	D-alanyl transfer protein DltB	0.3	0.7
	SXYL_01988	*dltC*	D-alanine—poly(phosphoribitol) ligase subunit 2	0.2	
	SXYL_01987	*dltD*	D-alanyl-lipoteichoic acid biosynthesis protein DltD	0.2	0.7
**F: NUCLEOTIDE TRANSPORT AND METABOLISM**					
	SXYL_02117	*nrdF*	Ribonucleoside-diphosphate reductase beta chain	1.7	3.7
	SXYL_02119	*nrdI*	Protein NrdI	1.6	3.1
	SXYL_00150	*nrdG*	Anaerobic ribonucleotide reductase activating protein		0.3
**H: COENZYME TRANSPORT AND METABOLISM**					
	SXYL_02415	*folB*	Dihydroneopterin aldolase	1.7	1.3
	SXYL_01872	*folD*	Bifunctional protein FolD	2.4	1.4
	SXYL_02414	*folK*	2-amino-4-hydroxy-6-hydroxymethyldihydropteridine pyrophosphokinase	1.4	
	SXYL_02416	*folP*	Dihydropteroate synthase	1.7	
**L: REPLICATION, RECOMBINATION AND REPAIR**					
	SXYL_01397	*nth*	Endonuclease III	1.4	
	SXYL_01796	*uvrC*	UvrABC system protein C	1.8	1.4
**K: TRANSCRIPTION, REGULATORS**					
	SXYL_00975	*perR*	Peroxide-responsive repressor PerR	2.0	
	SXYL_00784	*czrA*	Zinc and cobalt transport repressor CzrA		2.1
	SXYL_01275	*hrcA*	Heat-inducible transcription repressor HrcA	0.7	
	SXYL_01250	*greA*	Transcription elongation factor GreA		0.5
	SXYL_01657	*fapR*	Transcription factor FapR	0.6	0.7
	SXYL_01129	*ccpA*	Catabolite control protein A	1.8	

Concomitant with up-regulation of genes involved in iron acquisition, a gene involved in iron storage, *fntA* which encodes ferritin, was up-regulated in *S. xylosus* in the presence of RNS (Table 1). Ferritins are ubiquitous iron-storage proteins which can also protect against metal toxicity and oxidative stress (Andrews, 1998). They have been well-characterized in *S. aureus* and *Staphylococcus epidermidis* (Horsburgh et al., 2001a; Morrissey et al., 2004).

**Figure 3:**
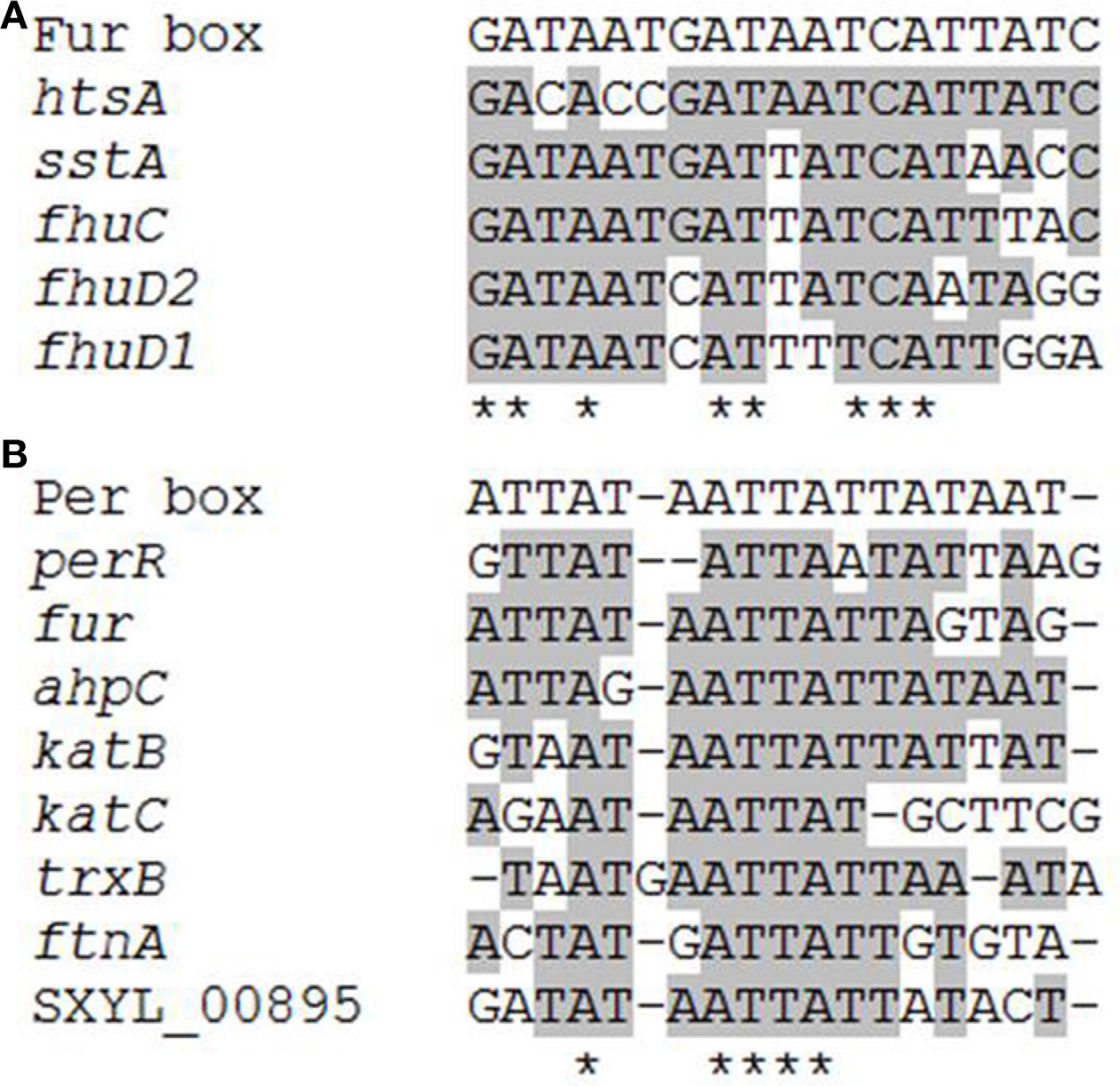
**Alignment of the putative Fur boxes (A) and PerR boxes (B) identified in the *Staphylococcus xylosus* C2a genes coding for iron homeostasis and antioxidant properties**. ^*^Conserved position.

**Figure 4:**
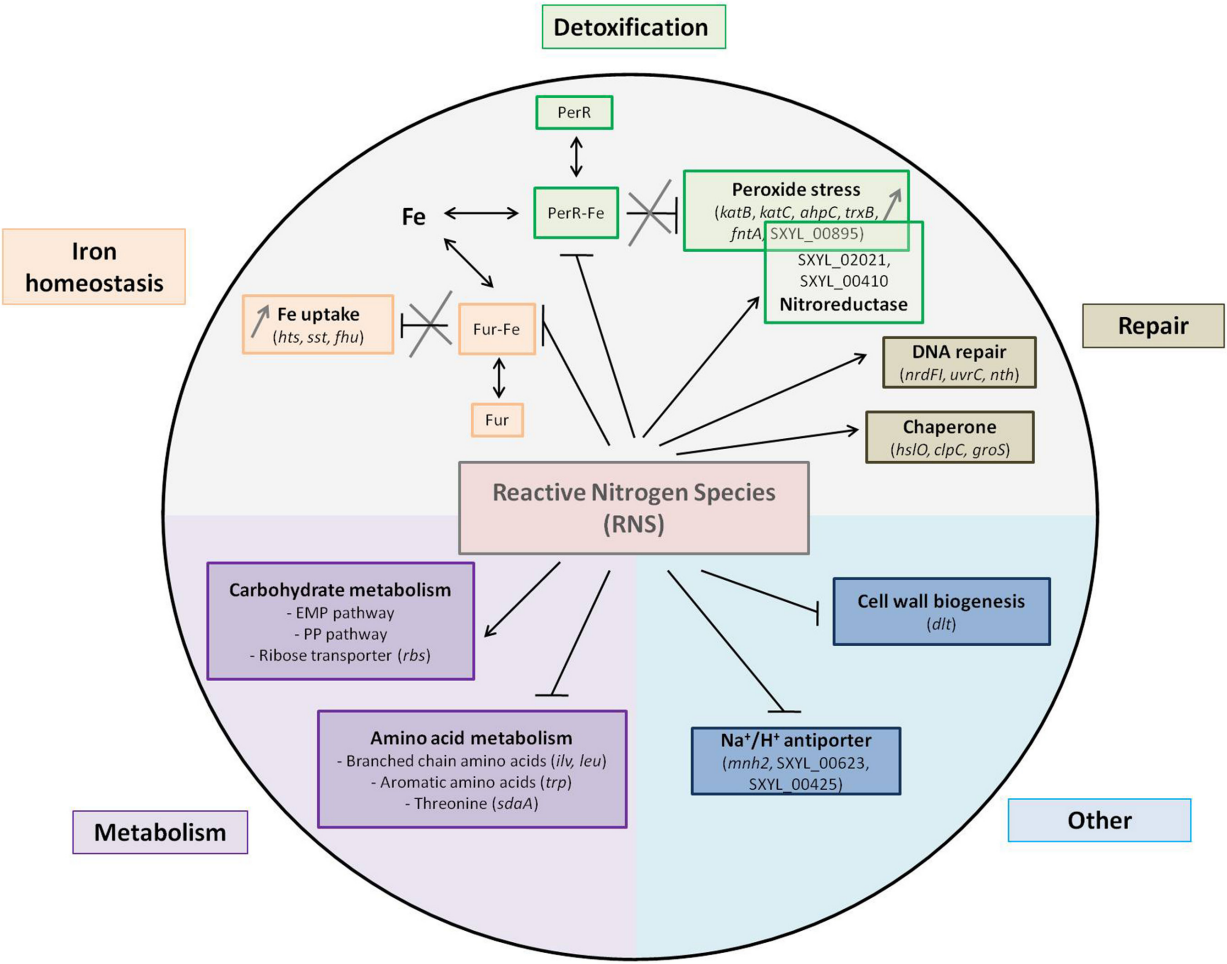
**Summary of the main pathways and regulations affected by nitrosative stress in *Staphylococcus xylosus* in a meat model**.

Iron is an essential nutrient, but as high levels of free intracellular ferric iron are toxic to cells, the amount of free iron has to be strictly regulated. In many bacteria, the ferric uptake regulator (Fur) functions as a transcriptional repressor for iron homeostasis. When complexed with iron, Fur regulates the transcription of genes by binding a consensus sequence known as the Fur box within the promoter region (Morrissey et al., 2004; Sheldon and Heinrichs, 2012). Genes involved in iron acquisition are repressed by Fur and thus Fur boxes controlling the expression of these genes in *S. aureus* have been described (Sheldon and Heinrichs, 2012). We have looked for Fur boxes in *S. xylosus* genes coding for iron acquisition by using the inverted repeat sequence of 19-bp described for *S. aureus*. We found putative sequences located close to a 35 bp site within the promoter region for *htsA, sstA, fhuC, fhuD1, fhuD2* with nucleotide identity to the *S. aureus* Fur box consensus sequence (Figure 3). Fur protein functions as a repressor. Under iron-replete conditions, Fe^2+^ binds to Fur and the Fur-Fe^2+^ homodimer binds to the Fur box sequence and inhibits the transcription (Hassan and Troxell, 2013). With iron deprivation, there is an increase in the transcription of genes involved in iron uptake. In meat, a lot of iron sources are available: ferritin, myoglobin, transferrin, and hemoglobin, which raises the question of why *S. xylosus* senses an iron deficiency environment in the presence of RNS. Fur can sense not only iron but also an oxidative environment (Fillat, 2014). It has been demonstrated that NO and peroxide can modulate Fur activity (Spiro, 2007). The direct interaction of NO with iron center of Fur leads to the inhibition of Fur activity resulting in the induction of iron-regulated genes by NO in *E. coli* (D'Autréaux et al., 2002; Mukhopadhyay et al., 2004). NO can react with iron in Fur-Fe and abolish DNA binding and thus induce genes repressed by Fe-Fur in *S. aureus* (Richardson et al., 2006). Thus, we hypothesized that NO from nitrite can inactivate Fur, thus derepressing the regulon, and consequently genes involved in iron intake are up-regulated in *S. xylosus* (Figure 4).

### Antioxidant defenses and the PerR regulon

The nitrosative stress can be augmented by intracellular iron, which can react with H_2_O_2_ by Fenton chemistry giving rise to damaging hydroxyl radicals (HO^·^). *S. xylosus* responded to this stress by the regulation of 12 genes involved in protection and detoxification mechanisms (Table 1, Figure 4). Among these genes, three encode catalases with *katA* down-regulated and *katB* and *katC* up-regulated. Most staphylococci are catalase-positive with the majority, like *S. aureus*, having a single catalase (Gaupp et al., 2012). *S. carnosus* has two catalases (Rosenstein et al., 2009). Some strains of *S. xylosus, Staphylococcus saprophyticus* and *Staphylococcus equorum* have been reported to have two catalases (Blaiotta et al., 2010). The *S. xylosus* strain C2a has three catalases. The transcription of *katA* in this strain is induced upon entry into the stationary phase, by oxygen and by hydrogen peroxide, whereas iron and manganese have a repressive effect (Barrière et al., 2002). KatB and KatC were identified in the cell envelope of this strain with KatC exhibiting similarity with KatE of *Bacillus subtilis* (Planchon et al., 2009). In addition, gene *ahpC* encoding alkyl hydroperoxide reductase, *trxB* encoding thioredoxin reductase and *bcp* for bacterioferritin comigratory protein were all up-regulated. In *S. aureus*, catalase detoxifies H_2_O_2_ while AhpC confers resistance to other ROS (Gaupp et al., 2012). After NO stress, KatA and AhpF were overexpressed in *B. subtilis* but not in *S. aureus* (Hochgräfe et al., 2008). Bacterioferritin comigratory protein (Bcp) functions as an iron chelator and is homologous to a thioreductase-peroxidase and as such is likely involved in the thiol-dependent reduction of peroxides (Gaupp et al., 2012). As described for other bacteria, the thioredoxin system of *S. xylosus* comprises thioredoxin (*trxA*) and thioredoxin reductase (*trxB*). Transcription of these two genes is increased in response to diamide, H_2_O_2_, heat, salt or ethanol stress in *B. subtilis* and in *S. aureus* (Gaupp et al., 2012). Thioredoxins are major contributors to oxidative resistance by facilitating the reduction of H_2_O_2_, and by scavenging HO^·^ (Gaupp et al., 2012). They help maintain protein thiols in their reduced form and thus the reduced state of the cytoplasm. Moreover, to respond to the nitrosative stress, the gene *bsaA* encoding glutathione peroxidase, which catalyzes the reduction of H_2_O_2_, and the gene SXYL_00570, which encodes ferredoxin-NADP reductase, were up-regulated in *S. xylosus*. In parallel to the up-regulation of *trxB*, the gene *msrA1* encoding a peptide methionine sulfoxide reductase (Msr) was up-regulated in *S. xylosus* in the presence of RNS (Table 1). Msr catalyzes the reduction of free and bound methionine sulfoxide [Met-(S)-SO; Met-(R)-SO] (Moskovitz, 2005). The catalytic process, with thioredoxin as reducing agent, includes the production of a sulfenic intermediate with concomitant release of the repaired methionine and then a recycling step where oxidized Msr is reduced to its active form (Ezraty et al., 2005). The oxidized S form of methionine is reduced by the enzyme MrsA while the R form is reduced by MsrB. *S. aureus* contains three paralogs of *msrA* (*msrA1, msrA2, msrA3*) and a single *msrB* gene (Singh and Moskovitz, 2003; Ezraty et al., 2005). Similarly, *S xylosus* contains three *msrA* genes and one *msrB*. In *S. aureus*, MsrA1 is the major contributor to tolerance of oxidative stress (Singh and Moskovitz, 2003).

Furthermore, two genes (SXYL_00895; SXYL_02021) encoding two probable nitroreductases and one gene (SXYL_00410) encoding a putative NAD(P)H nitroreductase were overexpressed in *S. xylosus* grown in the presence of RNS (Table 1, Figure 4). Nitroreductases have been classified into three classes: class A represented by *E. coli* NfsA and including *S. aureus* NfrA, class B represented by *E. coli* NfsB and class C including *S. aureus* NtrA (Tavares et al., 2009). The *S. aureus* NfrA is a flavin mononucleotide-dependent NADP oxidase involved in the oxidative stress response (Streker et al., 2005). In addition, this protein showed nitroreductase activity and weak disulfide reductase activity. The transcription of *nrfA* was strongly induced by nitrofurantoin, thiol-specific oxidant diamide and high concentrations of hydrogen peroxide, and a PerR box was identified (Streker et al., 2005). A putative PerR-binding site upstream of SXYL_00895 has been identified (Figure 3). As for *S. aureus*, this nitroreductase may play a role in the nitrosative stress response and help to maintain the thiol-disulfide balance. Moreover, SXYL_02021 shows a high degree of similarity with NtrA. NtrA has an S-nitroso-glutathione reductase activity involved in nitrosative metabolism (Tavares et al., 2009). Our results led us to suppose that in *S. xylosus* three nitroreductases could be involved in the response to nitrosative stress.

The gene *per* encoding the transcriptional regulator PerR, which belongs to the Fur family, was up-regulated in the presence of RNS in *S. xylosus* (Table 1). As a member of the Fur family, the activity of PerR is dependent on metal ions (Horsburgh et al., 2001b). In *B. subtilis*, PerR carries structural zinc and its DNA-binding activity is enhanced by Fe (II) or Mn (II) (Gaupp et al., 2012). PerR, containing Fe or Mn, functions as a transcriptional repressor by binding to a consensus DNA sequence called the PerR Box (Horsburgh et al., 2001a; Gaupp et al., 2012). *S. aureus* PerR regulates genes for antiperoxidative enzymes and iron storage proteins and represses the *fur* gene, which controls iron homeostasis. We looked for the PerR box in *S. xylosus* genes involved in iron acquisition and storage and antioxidant properties by using the consensus sequence of 17-bp described for *S. aureus* (Horsburgh et al., 2001a,b). We found a putative PerR-binding site upstream of the coding sequences of *katB, katC, ahpC, trxB*, and *fntA* (Figure 3). No PerR box was found upstream of the *katA* gene, which was down-regulated in response to RNS, the expression of *katB* and *katC* being up-regulated under the probable control of PerR. PerR boxes were also found in the promoter regions of *fur* and *perR*, suggesting autoregulation (Figure 3). The nitrosative stress in *S. xylosus* resulted in a response similar to that for peroxide stress in *S. aureus*, except that transcription of *perR* was also increased. PerR has been identified as a peroxide-sensing protein (Horsburgh et al., 2001a). It is only the Fe (II)-bound PerR that shows pronounced sensitivity to low levels of peroxide (Lee and Helmann, 2006). In the presence of H_2_O_2_, the iron in PerR leads to formation of a hydroxyl radical which oxidizes histidines at the binding site and prevents DNA binding (Fillat, 2014). In *S. aureus*, addition of H_2_O_2_ results in increased transcription of most of the PerR regulon except for *fur* and *perR* itself (Horsburgh et al., 2001a). As already mentioned above, Fur was inactivated by nitrosative stress and consequently transcription of the Fur regulon was increased, resulting in iron uptake. Elevated iron concentration could affect the distribution between the two forms of PerR, PerR-Fe, which is sensitive to oxidation, and the less sensitive PerR-Mn (Horsburgh et al., 2001a), and thus will induce the PerR regulon thereby keeping the system going (Figure 4). It is worth noting that the gene *mntH* encoding the transport of manganese was up-regulated in *S. xylosus* in the presence of RNS. Mn (II) is important for detoxification because it has a higher reduction potential than Fe (II), and it can function as an antioxidant, unlike iron, which is a pro-oxidant metal (Gaupp et al., 2012).

### Carbohydrate transport and metabolism

Glucose (0.5%) added to the two meat batches supported the growth of *S. xylosus* for up to 24 h. Glycogen from meat could therefore be a source of glucose and ATP from meat could be a source of ribose and contribute to energy production (Chaillou et al., 2005). Indeed, genes encoding enzymes involved in the Embden-Meyerhof-Parnas (EMP) and the pentose phosphate (PP) pathways were modulated at t_24h_ and t_72h_ in the presence of RNS (Table 1) (Figure 4). Each of these pathways has its own function. The EMP pathway (combined with the tricarboxylic acid cycle) is the general route for glucose catabolism and energy formation in the cell, while the PP pathway plays a crucial role in redox metabolism (Kleijn et al., 2006). Four genes (SXYL_02255, SXYL_00253, SXYL_00278, SXYL_00626) encoding phosphotransferase systems (PTS) that catalyze the concomitant uptake and phosphorylation of carbohydrates were modulated (Table 1). The two genes paralogs (SXYL_02255, SXYL_00626) coding for a PTS for alpha-glucoside and the gene (SXYL_00278) coding for a fructose-specific PTS system were up-regulated at t_24h_, while the gene SXYL_00253 was down-regulated (Table 1). Five other genes (*malA, pgi, fbp, iolJ, fda*) of the EMP and/or PP pathway were also up-regulated (Table 1). Genes encoding enzymes involved in the uptake and metabolism of two substrates (ribose, arabinose) were modulated by RNS (Table 1). Three of them coding for the ribose ABC transporter (*rbsB, rbsC, rbsD*) were up-regulated at t_24h_ (Figure 4). For arabinose metabolism, the genes *araT, araA* and *araD* were down-regulated in the presence of RNS (Table 1). These genes belong to a cluster of five genes (*araRBDAT*) that encode proteins for uptake of arabinose and conversion to D-xylulose-5P, which can enter the pentose phosphate pathway (Siezen et al., 2008; Passerini et al., 2013). This cluster has not yet been characterized in *Staphylococcus*. Moreover, the presence of RNS modulated the transcription of two genes (*araB1, araB2*) encoding ribulokinases involved in the pentose interconversion (Table 1).

Because of the importance of these two metabolic pathways, staphylococci have regulators that sense the availability of these metabolites. Nitrosative stress can alter enzymatic activity resulting in changes in metabolite concentrations and redox balance; these changes will induce the response of these regulators. One form of carbon catabolite repression relying on a transcriptional regulator termed CcpA was up-regulated in *S. xylosus* in the presence of RNS at t_24h_ (Table 1). This regulator requires the co-repressor P-Ser-HPr to bind efficiently to its operator sequence *cre* (catabolite responsive element) (Brückner and Titgemeyer, 2002). In our study, the gene coding for the co-repressor was not modulated.

### Amino acid transport and metabolism

Transcript levels of eight genes involved in the metabolism of branched-chain amino acids (*ilv, leu* genes co-localized) and one involved in their transport (*brnQ-1*) were found to be down-regulated in *S. xylosus* in the presence of RNS (Table 1, Figure 4). In fermented sausages, catabolism of branched-chain amino acids (leucine, isoleucine, valine) results in methyl aldehydes, alcohols and acids involved in flavor (Stahnke, 1995). *S. xylosus* plays a fundamental role in this metabolism (Stahnke, 1999). Nitrate or nitrite was found to inhibit the degradation of leucine by *S. xylosus* in meat extract and in laboratory media (Moller et al., 1998; Olesen et al., 2004). This inhibition could be explained by the down-regulation of several genes involved in this metabolism.

Five genes involved in glycine/serine/threonine metabolism were also modulated (Table 1). Three of these genes organized in a cluster (*gcvT, gcvPA, gcvPB*) were up-regulated while two genes (*sdaAA1, sdaAB1*) coding for two subunits of the enzyme L-serine dehydratase, which belongs to the iron sulfur-dependent L-serine dehydratase family, were down-regulated in *S. xylosus* in the presence of RNS (Figure 4).

Sulfur-containing amino acids such as methionine and aromatic amino acids (tryptophan, phenylalanine, and tyrosine) can be oxidized during nitrosative stress (Gaupp et al., 2012). In our conditions, one gene (SXYL_00012) encoding a protein involved in methionine synthesis and 12 genes coding for proteins involved in aromatic amino acid synthesis were modulated (Table 1). Seven genes (*trpA* to *trpG*) encoding enzymes involved in tryptophan synthesis were organized in one cluster in *S. xylosus* as reported for most Gram-positive bacteria (Gutierrez-Preciado et al., 2005). Six of these genes and the gene SXYL_01128 encoding a DAHP synthase-chorismate mutase were down-regulated in our conditions (Table 1). Nitrosative stress can also modulate the expression of genes involved in sulfur-containing amino acids synthesis (Figure 4).

Transcript levels of 11 genes encoding enzymes involved in the urea cycle and metabolism of amino groups were modulated in *S. xylosus* in the presence of RNS (Table 1). Four of them (*rocD1, argC, argB*, SXYL_02450) coding for enzymes involved in the metabolism of ornithine were down-regulated. The gene *ansA* coding for an enzyme involved in aspartate metabolism, which can fuel the urea cycle, the gene *argG* coding for a urea cycle enzyme and six genes (*ure*) coding for urease were up-regulated. Urease activity generates ammonium which is a source of nitrogen, but ammonium ions can also alkalinize the cytoplasm and contribute to the ΔpH component of the membrane potential, or the ammonia molecule can diffuse from the cells (Jose et al., 1994; Burne and Chen, 2000). Urease activity can furnish *S. xylosus* with ammonium and protect it against the acid pH of the meat model (pH 5.7), but in our conditions there was no difference in pH with or without nitrate and nitrite.

Transcript levels of four genes (*ampA*, SXYL_01931, SXYL_01348, *dapE*) encoding peptidases with metallopeptidase activity were found to be up-regulated (Table 1). The gene SXYL_01931 encodes an oligoendopeptidase F. The *S. aureus* corresponding gene was up-regulated after vancomycin treatment or after exposure to AgNO_3_, which caused oxidative stress (Kuroda et al., 2003; Smith et al., 2012). The cytosol aminopeptidase encoded by the gene *ampA* (DQ498891) has already been identified in the cell envelope fraction of *S. xylosus* (Planchon et al., 2007). This aminopeptidase binds manganese ions, and it is noteworthy that the expression of the gene *mntH* involved in manganese transport was also up-regulated.

Osmotolerance is one of the characteristic features of staphylococci. They can import osmoprotectants by several transport systems (Kuroda et al., 2005). In our study, the cluster *opuC* (*opuCABCD*) encoding a glycine betaine/carnitine/choline ABC transporter was down-regulated at t_24h_, but the *opuCC* and o*puCD* genes were up-regulated at t_72h_ (Table 1). This late up-regulation may indicate a modification of the osmotic pressure. In contrast, the gene *opuD1* coding for an uptake system of glycine betaine was down-regulated over time in the presence of RNS (Table 1). The down-regulation of *opuD* has already been reported in *S. aureus* subject to combined oxidative and nitrosative stresses (Nobre and Saraiva, 2013). The transporters OpuC and OpuD may respond differently to osmotic environments, but it is not clear why these genes are modulated in our conditions.

### Energy production and conversion

The gene *atpD* coding for one subunit of ATPase synthase was down-regulated at t_24h_ in the presence of RNS (Table 1). ATPase synthase genes were down-regulated in *Desulfovibrio vulgaris* after nitrite stress (He et al., 2006). These results suggest that the electron flow was likely shifted from respiratory phosphorylation to nitrate reduction (He et al., 2006).

In our study, five Na^+^/H^+^ antiporter systems were modulated by addition of nitrate and nitrite: either encoded by single genes (SXYL_00623, SXYL_00425, *nhaC*) or by clusters of genes (*mnh*) (Table 1, Figure 4). Sodium proton antiporters are ubiquitous membrane proteins involved in cell energetics and play a primary role in the homeostasis of intracellular pH and cellular Na^+^ content, the establishment of an electrochemical potential of Na^+^ and cell volume. Nine different Na^+^/H^+^ antiporters have been identified in Gram-positive and Gram-negative bacteria (Majernik et al., 2001; Lorca et al., 2007). Bacteria have several antiporters catalyzing ostensibly similar reactions and the contribution of each antiporter is not yet established. Antiporter Mnh belongs to the cation/proton antiporter-3 (Mrp) family (Swartz et al., 2007). Mrp antiporters require six or seven genes for full activity and are highly conserved. Two Mnh antiporters encoded by two operon paralogs (*mnh, mnh2*) of seven genes have been described in *S. aureus* (Hiramatsu et al., 1998; Swartz et al., 2007). Similarly, seven genes (*mnhA* to *mnhG*) are also present in the two *S. xylosus mnh* cluster paralogs. In the *mnh* cluster, only *mnhG* was up-regulated in *S. xylosus* at t_72h_, while in the *mnh2* cluster, five genes (*mnh2ADEFG*) were down-regulated at t_24h_ (Table 1, Figure 4). The expression of *nhaC* coding for the Na^+^/H^+^ antiporter NhaC of *S. xylosus* was up-regulated in the presence of RNS at t_24h_ and t_72h_ (Table 1). The modulation by RNS of genes encoding Na^+^/H^+^ antiporters has not yet been described. But stresses such as pH, salt, temperature and interplay between these stresses can regulate the level of expression and activity of these antiporters (Padan et al., 2005). Further studies are required to define the specific mechanisms that modulate the efficacy of the antiporters and in some cases their capacity to catalyze multiple activities.

### Cell wall/membrane biogenesis

Transcript levels of four genes (*dlt*A, *dltB, dltC, dltD*) involved in D-alanylation of teichoic acids were down-regulated in *S. xylosus* in the presence of RNS (Table 1, Figure 4). These four genes are organized in an operon (AF032440) (Peschel et al., 1999). Esterification of D-alanine to teichoic acids has pleiotropic effects such as regulation of autolysins, binding of cations (Na^+^, Mg^2+^, Ca^2+^) to the cell envelope and resistance to antimicrobial cationic peptides and proteins (Neuhaus and Baddiley, 2003; Schneewind and Missiakas, 2014). In *S. aureus*, the degree of D-alanylation depends on the environmental conditions, and decreases with increase in pH, temperature or salt (Neuhaus and Baddiley, 2003). Moreover, *dlt* expression is repressed in response to Na^+^, Mg^2+^, and Ca^2+^ (Koprivnjak et al., 2006). It is noteworthy that in our conditions the gene *mgtE* coding for a protein involved in magnesium transport and the gene *nhaC* coding for an antiporter involved in the uptake of Na^+^ were up-regulated in *S. xylosus*.

### DNA synthesis and repair

Transcript levels of three genes encoding ribonucleoside reductases (RNRs) were modulated (Table 1). Two of them (*ndrI, nrdF*) were up-regulated, while *nrdG* was down-regulated. RNRs are enzymes essential for the synthesis of deoxyribonucleotides, the precursors of DNA synthesis or repair (Figure 4). They catalyze the reduction of the four ribonucleotides to maintain a pool of deoxyribonucleotides during the cell cycle. Three classes of RNRs are known (Rabinovitch et al., 2010). *S. aureus* contains Class Ib and class III RNRs, which are essential for aerobic and anaerobic growth, respectively (Masalha et al., 2001). Class III RNRs are encoded by *nrdD* and *nrdG* genes in *S. aureus* (Masalha et al., 2001). Similar organization is found in the genome of *S. xylosus*, but *nrdD* is truncated, i.e., a pseudogene. It is worth noting that two truncated parts of *nrdD* (SXYL_00148; SXYL_00149) were transcriptionally active and were also down-regulated, as mentioned for *ndrG*. The class Ib NrdEF is encoded by the *nrdE* and *nrdF* genes, which form an operon containing a third gene *nrdI* which overlaps with *nrdE* in *S. aureus* (Masalha et al., 2001). We observed the same cluster of three genes *nrdIEF* in the *S. xylosus* genome. In *S. aureus*, NrdEF are able to function with thioredoxin reductase as the donor of hydrogen (Rabinovitch et al., 2010). In our study, the expression of the gene *trxB* encoding thioredoxin reductase was up-regulated (Table 1). In *E. coli*, expression of the *nrdHIEF* operon is triggered in response to oxidative stress (Monje-Casas et al., 2001). This induction is mediated by the inactivation of Fur (Martin and Imlay, 2011). In our study, as already stated, nitrosative stress inactivated Fur in *S. xylosus*, and a similar mechanism could explain the up-regulation of *nrdI* and *nrdF* (Figure 4).

Four genes (*folD, folB folK, folP*) encoding proteins involved in the synthesis of folate were up-regulated in *S. xylosus* in the presence of RNS (Table 1). As *S. xylosus* C2a is able to grow in a minimal medium including only three vitamins (thiamine, nicotinic acid, and biotin) (Fiegler and Brückner, 1997), we can suppose that it has the capacity to synthesize folate. Folate is required as a cofactor in one-carbon-atom transfer reactions, including methionine, purine, and thymine syntheses, and it has been proposed as an antioxidant that scavenges peroxyl, azide and hydroxyl radicals (Huang et al., 2004; Rossi et al., 2011).

Two genes, *nth* encoding an endonuclease type III and *uvrC* encoding UvrABC system protein C, involved in DNA excision repair were up-regulated in *S. xylosus* in the presence of RNS (Table 1, Figure 4). In *E. coli*, pyrimidine lesions generated by free radical HO are recognized by endonuclease types III and VIII (Wallace, 1988). The complex UvrABC is involved in nucleotide excision and repair, and the genes *uvrABC* are reported in *S. aureus* (Ambur et al., 2009) and also present as a cluster in *S. xylosus*.

### Post-translational modification, protein turnover, chaperones

Chaperones and ATP-dependent proteases play a major role in bacterial survival under conditions of stress where proteins tend to unfold and aggregate. In our conditions, nitrosative stress leads to an increase in the expression of *hslO* (heat shock protein 33 homolog), *clpC* (ATP-dependent Clp protease ATP-binding subunit clpC) and *groS*, (Table 1, Figure 4). The heat shock protein Hsp33 was first found to be induced by heat shock, but it has been discovered that it requires both unfolding as well oxidative conditions to become active (Ilbert et al., 2007). Hsp 33 contains a bound Zn^2+^ ion coordinated by four cysteines, and under oxidizing conditions two disulfide bonds are formed involving the reactive cysteines, while under reducing conditions zinc is bound to the reactive cysteines and the protein is inactive. It is worth noting that the gene *czrA* encoding a zinc and cobalt repressor was up-regulated in *S. xylosus* (Table 1), as already described for *S. aureus* during nitrosative stress (Nobre and Saraiva, 2013). The gene *clpC* has been shown to be induced in heat-shocked cells in *S. aureus* (Frees et al., 2004). It is noteworthy that the expression of the heat-inducible transcription repressor *hcrA* was down-regulated in *S. xylosus* in the presence of RNS. In *S. aureus*, HrcA regulator is required to repress transcription of *groS* and is modulated by the thiol-specific oxidant diamide (Wolf et al., 2008).

## Conclusions

In this study, determination of the global gene expression of *S. xylosus in situ* in meat in the presence of nitrate and nitrite at the levels used in the meat industry has allowed us to unravel the adaptive response of this bacterium to nitrosative stress. *S. xylosus* counteracts nitrosative stress by developing several oxidative stress resistance mechanisms such as modulation of the expression of genes involved in iron homeostasis mostly under the control of Fur, detoxifying enzymes mostly under the control of PerR, and DNA and protein repairs. Nitrosative stress could alter Fe-S clusters and sulfur-containing proteins and *S. xylosus* adapts to these changes in metabolite concentrations by modulating the expression of several genes involved in carbon and nitrogen metabolism, energy production and cell wall biogenesis. Our results offer the sole data set for the response of a food-associated bacterium when exposed to nitrate and nitrite in meat. Despite the low concentration of nitrate and nitrite used, a significant change in the *S. xylosus* gene expression was observed. The strategies to reduce the input of nitrate and nitrite in meat products should also take into account the potential impact on bacterial starter cultures.

### Conflict of interest statement

The Review Editor Yves Le Loir declares that, despite being previously involved in a joint funding project with the author Régine Talon, the review process was handled objectively and no conflict of interest exists. The authors declare that the research was conducted in the absence of any commercial or financial relationships that could be construed as a potential conflict of interest.
