# Supplementary material for: Transcriptomic analysis of Staphylococcus xylosus in the presence of nitrate and nitrite in meat reveals its response to nitrosative stress
Source: Front Microbiol. 2014 Dec 15;5:691. doi: 10.3389/fmicb.2014.00691 (PMC4266091; doi:10.3389/fmicb.2014.00691)
Supplement: Supplementary file 1 [file Table1.PDF]

Supplementary Table 1. Targeted genes of *Staphylococcus xylosus* for the validation of microarray data by qPCR: expression at t_{24h} or t_{72h} in meat in the presence of nitrate and nitrite.

Gene	Sequences of primers pairs for qPCR (5'-3')		Ratio of expression at indicated time as determined by:			
			Microarray		qPCR	
			24h	72h	24h	72h
rpsB	GCTAGAAGCAGGTGTTCACT	CCGCCATCTTCTGATACTTGT	0.5	0.5	0.5	0.5
queC	GGCTATCGTAGTGTTTAGTGGT	ATGTTGTTCTTCTGCAATGCG	0.4	0.5	0.6	0.6
pheT	TAACGTGCTTGCTGAACGTAT	TGGCTCTACTTCACCAATGTC	0.6	0.7	0.8	0.6
srtA	TATCCAGGACCTGCTACACC	TGCCTACTTTTGCCTCTGG	0.6	0.7	0.7	0.6
leuC	GCGACACTTGACCATAATGTG	CTGTTTCAGGTCCAACCATGT	0.2	0.2	0.4	0.4
ilvC	GGCTATGGATCTCAAGGACAC	ACAGGATATACTTCGAAGCCG	0.2	0.2	0.4	0.4
trpC	TGCTCGAACAGGGCTATTATG	GTGCTAAATCTCTACCTGGCA	0.5	0.3	0.7	0.5
metK	GTTGCTTGTGAGACGACAGTA	GCCATTGTCTGGCTATCGTA	0.4	0.3	0.5	0.4
nrdI	TGGTCACTGGAACGATAGGAT	TCTGAGATAGACCGTCCAGC	1.5	3.0	1.9	4.6
mntH	TCCGTCAATGCTCATGGTAAG	GCACCACCTTGCATAGATGT	1.4	2.2	1.5	3.9
ispD	TAGCAGGCGGTATTGGTTC	CCTTCGTATGCGAAATCCAC	1.5	0.5	2.4	0.7
rplJ	GTGGTCTAAGCGTTGCTGA	GTAGCAACTGCAGTAGGACC	0.6	-	0.6	-
dltD	GGTACTGGTGGTTCGACAG	AACTGATTGAGCTGTGCCT	0.3	-	0.2	-
proC	GTGCTGGAAATATGGCACATG	ATTGACGCCTAACTCATCAGC	1.5	-	1.5	-
gcvT	GATGGGCTATGCCAGTCC	ACTGTGCAGCTTCACTACC	3.3	-	6.0	-
aroD	TCGAACGAAGACTCAAGGTG	TTGCGATGCGCTGATTCTA	1.7	-	2.5	-
ureA	GCAGCAGATTTAGCTCGTAGA	CCTCCATTACATCATCCTCACC	3.1	-	3.9	-
argC	AGGTAGTGGTTATGGAGCGAT	GCGTTAAGTGACTCAAGTGAG	0.5	-	0.6	-
hutG	CTGGCACAACGTGATCTTTG	TCAGGACCTTCTTTAGCACC	1.9	-	2.1	-
deoD	TGTATTAATGCCAGGCGATCC	AATACTTGGTACGCCCATTCC	2.0	-	2.4	-
nagA	GGACGCTTCATTTGAAGGATT	TCCCAACAATACTAGCTGCAT	1.7	-	2.6	-
fruK	AGGAGGCTTTCCAGGACAA	GTGGACCAGGCGCATTAAT	0.6	-	0.6	-
SXYL_02582	ACGCCTGTAGCTCAAATAGTG	GGCTGCGAATTCTCTGGAAT	2.0	-	2.8	-
drp35	AGTGAGGTGCCTATTCTTGC	GAAGCAAAGCCCTTCAAGTTG	2.3	-	2.4	-
SXYL_00101	GCAGAAGAAGGTGCAAACG	GCGTTATTGACCAAGCCATC	2.0	-	8.5	-
fda	TGGTAGTACACCTAAAGCGC	TGATGAAACAATACGTGTACGC	2.2	-	2.6	-
folB	TTATCATGGCGCGTTACCA	GGCTTCCCTTCCATAATCGC	1.6	-	2.1	-
fabD	ATAGCTTAGGCGAGTATGCG	AACTTCCTCGTAATCCAAGCC	0.6	-	0.7	-
hslO	CGTGCATACAGTGCTTTGAC	CACCTTTCAACATAGCACCC	1.7	-	2.5	-
panD	CATAGAGCGAGAGTGACGG	ACCACTACCTCGTTCACCTT	0.6	-	0.6	-
SXYL_00607	ATGGGCACAGTGTCAATAGT	TTACGAGGTAATCGCCTGC	2.1	-	2.6	-
ahpC	CACAGCTAACGCTTATGATCC	CGAATGAGAAGTCTGCTGGG	-	2.0	-	2.6
ldh2	GCAAGTGGAGAAGCTCTTGAT	ACTCGCAGCAACCACAATAA	-	0.6	-	0.6
glpD	GCACAAGGTACAAGTTCACG	AAGCATACGTTCAGGTGTTGT	-	0.7	-	0.8
tyrA	ATGCAACACCCGTACAACA	TTGGATGGCCACCGATAAG	-	2.5	-	2.8
carA	ACGGCAATGACGGGTTATC	GGTTGGCTGCTGCTTTCT	-	0.5	-	0.5
guaB	GGCTAGACAAGGTGGACTTG	ACCCATCAATGCTTCTGCTT	-	1.5	-	1.5
bioB	GAAGACGCAAGTGTGGATACA	TGCCCACAATAGCCACAATT	-	0.4	-	0.3
