# Supplementary material for: Transcriptomic analysis of Staphylococcus xylosus in the presence of nitrate and nitrite in meat reveals its response to nitrosative stress
Source: Front Microbiol. 2014 Dec 15;5:691. doi: 10.3389/fmicb.2014.00691 (PMC4266091; doi:10.3389/fmicb.2014.00691)
Supplement: Supplementary file 2 [file Presentation1.PDF]

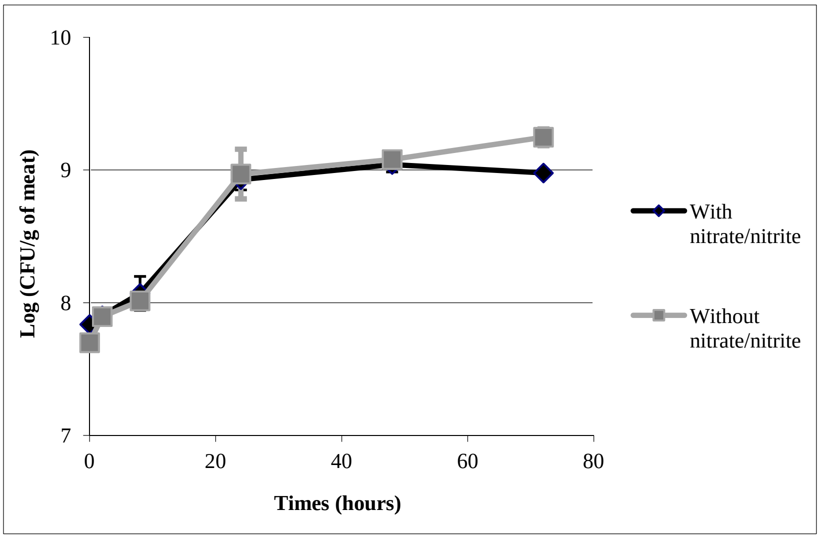

Supplementary Figure1. ***Staphylococcus xylosus*** **growth over time in the presence or absence of nitrate and nitrite in the meat model.**
